# Assessing the Use of Standardized Outcome Measures for Stroke Rehabilitation among Physiotherapists in Ghana

**DOI:** 10.1155/2020/9259017

**Published:** 2020-07-01

**Authors:** Seth Kwame Agyenkwa, Cosmos Yarfi, Adjoa Nkrumah Banson, Woyram Abla Kofi-Bediako, Ulric Sena Abonie, Seth Kwadjo Angmorterh, Eric Kwasi Ofori

**Affiliations:** ^1^Department of Physiotherapy and Rehabilitation Sciences, University of Health and Allied Sciences, Ho, Ghana; ^2^Department of Medical Imaging, University of Health and Allied Sciences, Ho, Ghana

## Abstract

**Background:**

The use of standardized outcome measures is an aspect of good clinical practice and essential to the rehabilitation of patients suffering from stroke. Literature reports regarding the extent of usage of outcome measures in stroke rehabilitation by physiotherapists globally are inconsistent. In addition, the patronage of outcome measures in stroke rehabilitation in low-resourced countries is uncertain.

**Objective:**

This study was conducted to assess the current practice of physiotherapists in Ghana regarding the use of standardized outcome measures in the rehabilitation of stroke patients.

**Method:**

A descriptive cross-sectional survey, was used involving 105 registered physiotherapists in Ghana. A 35-item adapted questionnaire was used to collect data on some commonly used outcome measures and frequency of use by physiotherapists for stroke patients.

**Results:**

A total of 55 (52.4%) physiotherapists did not use outcome measures in their clinical practice. Physiotherapists below 40 years of age use outcome measures (64.7%) more than those 41 years and above (6.7%). Physiotherapists working in public facilities in Ghana are more likely to use outcome measures (56.2%) than those in private facilities (16.2%). Physiotherapists who attend to 1-10 patients in a week used outcome measures more (32.4%) than physiotherapists who attend to more than 30 patients (3.8%) in a week.

**Conclusion:**

There is poor usage of outcome measures by Ghanaian physiotherapists, with more than half of the participants not using any standardized outcome measures for rehabilitation of patients in their practice. Physiotherapists who attends to fewer number of patients in a week are more likely to use outcome measures. There is the need for implementation of policy and guidelines on the use of outcome measures by the Allied Health Professions Council and the Ghana Physiotherapy Association.

## 1. Introduction

Outcome measures are assessments that measure change in patients' functioning, performance, or participation over time [1]. The use of a standardized assessment tool in stroke care is an important element of evidence-based rehabilitation [2], which has been widely documented [3]. Good clinical care involves monitoring patients' status through the appropriate use of outcome measures [4].

Outcome measures are known to inform clinical decisions such as planning treatment and setting realistic treatment goals [5]. The integration of outcome measures into clinical practice improves patient care and enhances communication with patients and their family on treatment goals [6]. Similarly, the use of stroke outcome measures is useful in monitoring the effectiveness of interventions and can serve as useful educational tools for patients and their families. In effect, outcome management facilitates communication between care settings and increases the efficiency of clinical practice among the multidisciplinary health professionals involved in the management of stroke [4, 7**].**

The effect and influence of physiotherapy treatment in the management of stroke can be assessed and established objectively by the use of outcome measures. Therefore, the use of outcome measures is strongly recommended worldwide [1]. The Intercollegiate Stroke Working Party [8] of London published clinical guidelines for the management of stroke and indicated that measurement of function is central to the rehabilitation process of stroke patients and that measurement of function is best achieved with the use of outcome measures. It is in line with this that the World Health Organisation (WHO) developed a number of assessment tools used by healthcare professionals to assess outcomes post stroke based on the International Classification of Function, Disability and Health [9].

In Ghana, the use of outcome measures by physiotherapists in most physiotherapy clinics is observed to be uncommon despite the importance of outcome measures in clinical practice. The Ghana Health Service (GHS), the body responsible for the implementation of national health policies in Ghana, and the Ghana Physiotherapy Association (GPA), the professional association of qualified and registered physiotherapists in Ghana, are yet to recommend some standardized outcome measures for the rehabilitation of stroke patients, despite its importance. There is therefore paucity of information on the extent of usage of outcome measures by physiotherapists for stroke rehabilitation in Ghana. The purpose of the study was to determine the extent of usage of standardized outcome measures among Ghanaian physiotherapists for stroke rehabilitation.

## 2. Methods

A cross-sectional survey, involving registered physiotherapists, were recruited for the study. The inclusion criteria was physiotherapists attending to stroke patients at least six months prior to the study. Physiotherapy students and interns on clinical placement and physiotherapists with less than one-year post qualification working experience were excluded from this study. To achieve the aim of the study, a 35-item questionnaire was developed based on previous studies [3, 10]; the questionnaire was pretested on five physiotherapists with similar characteristics as the participants of the study. The questionnaire consisted of two parts: part one captured information on the demographics of the participants such as age, sex, level of education, years of working experience, type of facility, working hours in a week, and the number of patients seen in a week. The second part of the questionnaire assessed the number, times, and frequency of usage of standardized outcome measures by the study participants. The pretesting was done to determine the face and content validity, and the reliability of the questionnaire developed. The feedback from the pretest was used to modify the wordings and structure of the questionnaire before implementing it in the main study. The face and content validity of the adapted questionnaire was found to be adequate by three experienced and expert physiotherapists in Ghana.

The study was advertised on the social media platforms of GPA with the contact details of the researchers so that physiotherapists interested in participating in the study could contact them. In all, 120 physiotherapists agreed to participate in the study, out of a total number of 165 physiotherapists working in Ghana [11]. The developed questionnaires were emailed to participants, who agreed to take part in the study, to fill and return to the researchers. This was after each participant had been provided with the participants' information sheet and consented to partake in the study. In addition to the email sent, an electronic-based data collection tool (Google forms) with an electronic link was sent to all physiotherapists working in various hospitals in Ghana. A reminder was sent to the participants every two weeks until the end of the study, when the completed questionnaires were not returned to the researchers.

The data collection lasted from January, 2019, to April, 2019. After the end of the study, one hundred and five completed questionnaires (105) were returned to the researchers.

All the data collected were checked carefully for accuracy and entered into Statistical Package for the Social Sciences (SPSS) version 25.0. The demographic information of the participants was analysed using frequencies, and percentages. The difference between the years of working experience and number of patients seen in a week by physiotherapists and use of outcome measures was determined using cross tabulations and the chi-squared test with a level of significance set at *p* < 0.05.

## 3. Results

A total of 105 physiotherapists participated in the study out of a potential 120 physiotherapists, representing a response rate of 87.5%. A greater number of the respondents, 96 (91.4%), were between the ages of 20 and 39 years. The remaining 9 (8.6%) were above 40 years but below 60 years of age. Majority 58 (55.2%) of the respondents were males, and females constituted 47 (44.8%) of the population. In terms of their level of education, 86 (81.9%) had a bachelor's degree and the remaining 19 (18.1%) had a master's degree. A greater percentage, 49 (46.7%), of the participants had working experience of 5–10 years, and only 1 (0.9%) of them had been practicing for over 20 years. A total of 77 (73.3%) of the participants worked at public hospitals, and 28 (26.7%) of participants worked in private hospitals. The demographic characteristics of the participants are presented in Table 1 below. The largest proportion of participants 67 (63.8%) worked for 31-40 hours in a week, with majority of them 39 (37.1%) attending to 11-20 patients in a week and 2 (1.9%) of them attending to more than 80 patients in a week. In terms of the number of stroke patients, 57 (54.3%) physiotherapists attend to 1-10 stroke patients in a week as shown in Table 1.

**Figure 1:**
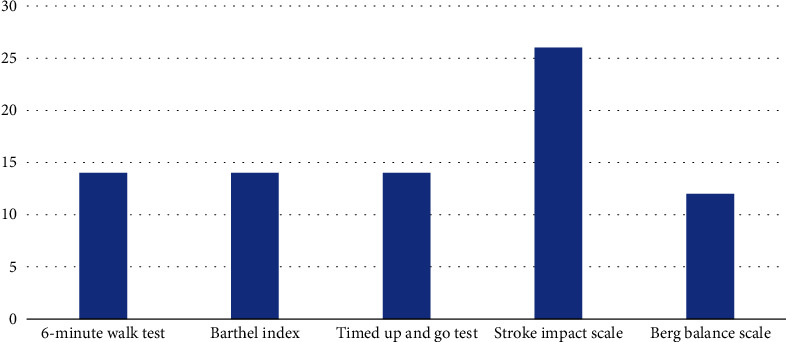
Top five commonly used outcome measures at facilities.

Over half of the participants, 55 (52.4%), reported that there were no recommended outcome measures in their facility for rehabilitation of stroke patients. The remaining 50 (47.6%) of participants have recommended outcome measures for use in their facilities: the top five outcome measures commonly used by physiotherapists in their facilities are Barthel Index 7 (14%), stroke impact scale 13 (26%), six-minute walk test 7 (14%), timed up and go test 7 (14%), and Berg balance scale 6 (12%) as shown in Figure 1 below.

**Figure 2:**
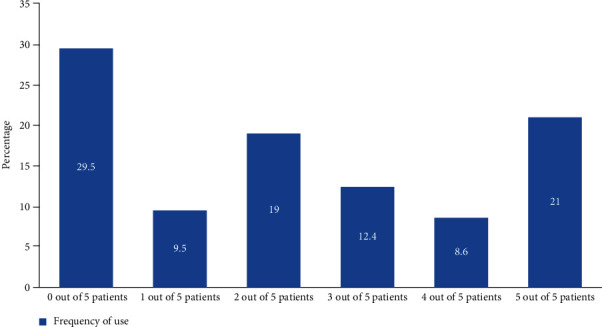
Frequency of usage of outcome measures by physiotherapists.

Majority of the participants 31 (29.5%) do not use any outcome measure for any of the stroke patients they managed, while 22 (21%) of them use outcome measures to evaluate the progress of 5 out of 5 patients seen, while 20 (19%) used the outcome measures for 2 out of 5 patients they see as shown in Figure 2.

The age, gender, level of education, and years of working experience of the study participants were found not to be statistically significant with the use of outcome measures. However, there was a significant association between the number of stroke patients seen a week by participants and the use of outcome measures with a *p* value of 0.013 as shown in Table 2.

There was a greater significance between the use of outcome measures and availability of recommended outcome measures for stroke in their facilities, with a *p* value of 0.0001. The use of outcome measures was high among participants where outcome measures were easily available and recommended in the facility, with a reported use of 46 (43.8%) among study participants. On the contrary, only 30 (28.6%) of the participants indicated the use of outcome measures for stroke patients where there were no recommended outcome measures in the facility.

## 4. Discussion

This study assessed the use of standardized outcome measures in the rehabilitation of stroke patients among physiotherapists in Ghana.

The results of this study showed that only 47.6% of physiotherapists in Ghana used recommended outcome measures (OMs) for the clinical management of patients. Similarly, 52.4% of the physiotherapists who participated in this study reported that there were no recommended OMs in their facility for rehabilitation of stroke patients, leading to nonusage of OMs in their clinical practice. Evidence in the literature shows that the degree of use of OMs varies between countries. For example, the results of this study reporting the absence and nonusage of OMs in 52.4% of physiotherapist in Ghana are consistent with similar studies from the United States of America (USA) and Egypt where 52% and 57% of physiotherapists, respectively, do not use OMs for stroke rehabilitation [12, 13]. However, very high usage of OMs has been reported in the United Kingdom (UK) and Saudi Arabia where 96% and 62% of physiotherapists, respectively, reported using at least one standardized OMs in stroke rehabilitation [14, 15]. The differences in the reported levels of usages of OMs could be attributed to the different levels of awareness of the usefulness of OMs in the clinical management of patients. In the current study, the use of OMs was higher in facilities where recommended OMs are available compared to facilities with no recommended OMs. There was a significant difference between the usage of outcome measures and the availability of recommended outcome measures in a facility.

The results of this study indicated that the commonly used OMs by physiotherapists in Ghana for stroke rehabilitation were the six-minute walk test, Barthel Index, time up and go test, stroke impact scale, and Berg balance scale. The stroke impact scale constituted the most used OM among physiotherapist in Ghana with 26% of the respondents using it in their practice. But this was in contrast with previous studies where the most frequently used OMs were the numeric pain rating scale (NPRS) and the visual analogue scale (VAS) for pain assessment and the Oswestry Disability Index (ODI) for low back pain [15–17]. However, Mabasa [18] reported Community Integration Questionnaire (CIQ), Maleka Stroke Community Reintegration Measure (MSCR), Barthel Index (BI), Quality of life (QoL) index, and the Rivermead Mobility Index as the top five OMs used by community physiotherapists in South Africa for their clinical practice. The wide variations in the usage of OMs found in these studies and the current study may be attributed to the lack of consistency and the purpose of use of OMs for clinical practice among physiotherapists. Another reason could also be that the current study was limited to the use of OMs for stroke rehabilitation in Ghana.

The results of this study indicated that the use of OMs for stroke rehabilitation is greater among physiotherapists aged 40 years and below (64.7%) compared to 6.7% for physiotherapists aged 41 year and above. Similarly, male physiotherapists were more likely to use OMS (40%) than female physiotherapists (32.4%). It must be stated that the differences between the usage of OMs and the age and sex of the physiotherapists were not statistically significant. The results of this study also showed that physiotherapists with 1-10-year work experience are more likely to use OMs (82.9%) than physiotherapists with more than 15-year work experience (3.8%). The position that physiotherapists with more work experience are less likely to use OMs than their colleagues with less work experience is supported by a study conducted in the Netherlands [3]. In the current study, majority of the physiotherapists (91.4%) who participated in the study were below 40 years of age, with only 8.6% of them aged 40 years and above. Thus, majority of the physiotherapists who participated in the study were young adults, and over 55% of the participants being males. The findings of the current study indicate that 11% of the physiotherapists were above 40 years of age and over 80% were aged between 21 and 30 years. The high percentage of young physiotherapists could be because physiotherapy training in Ghana started about 20 years ago. These could have accounted for more male physiotherapists and younger physiotherapists using OMs as compared to older physiotherapists.

Physiotherapists with a bachelor's degree were more likely to use OMs (59.1%) than those with a postgraduate degree (13.3%). This finding can be explained by the fact that physiotherapists with a bachelor's degree form the vast majority (81.9%) of the study participants, with just 18.1% having a master's degree. The smaller number of physiotherapists with a master's degree is due to the absence of a postgraduate training in physiotherapy in Ghana, with all the physiotherapists with a master's degree having schooled abroad. This makes postgraduate studies expensive for young physiotherapists to be able to afford it. However, previous studies have shown that physiotherapists with a master's degree are more likely to use OMs in stroke rehabilitation compared to those with a bachelor's degree [15, 19].

The current study also found out that physiotherapists working in public facilities in Ghana are more likely to use OMs (56.2%) than those in private facilities (16.2%). The difference was, however, not statistically significant. The less usage of OMs in the private hospitals may be due to the engagement of lower care of physiotherapists. In addition, poor monitoring and audit of the physiotherapy services provided at private facilities may account for the low usage of OMs in private facilities. The finding that physiotherapists working in public facilities in Ghana are more likely to use OMs than those in private facilities is not peculiar to Ghana. Evidence exist in literature that this trend exists in other countries where physiotherapists working in private practice showed poor adherence to clinical practice guidelines on the management of stroke patients [3, 5].

In the current study, it was found that the number of stroke patients seen in a week by physiotherapists has an effect on the use of OMs for stroke management. Physiotherapists who attend to 1-10 patients in a week used OMs more (32.4%) than physiotherapists who attend to more than 30 patients (3.8%) in a week. From the study, 37.1% of the physiotherapists attend to between 11 and 20 patients in a week. The number of stroke patients seen in a week by physiotherapists was found to be statistically significant. This is consistent with a study by Mabasa et al., 2017, where 20.8% of the community physiotherapists attend to fewer than 35 patients in a week while 56.3% of the physiotherapists attend to between 35 and 40 patients in a week. The large number of patients seen in a week by the physiotherapists could be due to the low number of physiotherapists in Ghana as seen in many developing countries [20].

The Allied Health professions Council (AHPC), the regulatory body for the practice of allied health professions, and the Ghana Physiotherapy Association (GPA), the professional body of licensed physiotherapists in Ghana, must develop, publicise, and implement a policy on the use of OMs by Ghanaian physiotherapists. The AHPC and the GPA could liaise with heads of physiotherapy departments and experts in the field of rehabilitation to develop guidelines for stroke rehabilitation and indications of specific OMs to use. The implementation of these guidelines should be monitored by rehabilitation managers with quality assurance in terms of audits. These audits should be conducted periodically to reinforce the importance of OMs in assessment and management of stroke patients [18]. This will help enforce the use of OMs in the clinical management of stroke patients. The GPA should develop a training program on OMs and test its implementation, investigating the usage among newly trained physiotherapists. The training institutions in Ghana should introduce physiotherapy students early on the use of OMs during their training and clinical practicum to help encourage usage among entry-level physiotherapists.

### 4.1. Limitations

The study examined the self-reported use of OMs among Ghanaian physiotherapists in the last six months. This could have resulted in recall bias among the study participants. Another limitation of the study was the inadequate response from the participants on semistructured questions.

## 5. Conclusion

The use of standardized outcome measures is an important tool that can be give valuable information about the patient for health professionals and to help guide patient management. There was poor usage of outcome measures by Ghanaian physiotherapists, with more than half of the participants not using any standardized outcome measures for rehabilitation of patients in their practice. However, physiotherapists attending to fewer number of patients in a week are more likely to use outcome measures. There is the need for implementation of policy and guidelines on the use of outcome measures by the Allied Health Professions Council and the Ghana Physiotherapy Association. The GPA and AHPC should liaise with special interest groups to offer courses on OMs. The need to start a postgraduate training in physiotherapy in Ghana is long overdue; continuous professional education should be provided within the workplace as workshops for physiotherapists. Some OMs should be adopted and conceptualized to the Ghanaian setting for usage among physiotherapists in Ghana.

## Figures and Tables

**Table 1:** Sociodemographic and working profile characteristics of the participant.

Parameter	Frequency (*n*)	Percentage (%)
Total (*N*)	105	100
Age		
20-24	16	15.2
25-29	30	28.6
30-34	34	32.4
35-39	16	15.2
40-44	4	3.8
45-49	1	0.9
50-54	2	1.9
55-59	2	1.9
Gender		
Male	58	55.2
Female	47	44.8
Level of education		
Bachelors	86	81.9
Masters	19	18.1
Working experience		
1-4 years	38	36.2
5-10 years	49	46.7
11-15 years	14	13.3
16-20 years	3	2.9
Above 20 years	1	0.9
Type of facility		
Public	77	73.3
Private	28	26.7
Working hours per week		
1-10 hours	3	2.9
11-20 hours	3	2.9
21-30 hours	16	15.2
31-40 hours	67	63.8
Above 40 hours	16	15.2
Stroke patients seen in a week		
1-10	57	54.3
11-20	28	26.7
21-30	15	14.3
31-40	2	1.9
41-50	3	2.9
Other patients seen in a week		
1-10	17	16.2
11-20	39	37.1
21-30	22	21.0
31-40	8	7.6
41-50	10	9.5
51-60	3	2.9
61-70	1	1.0
71-80	3	2.9
Above 80	2	1.9

**Table 2:** Association between participants' characteristics and the use of standardized outcome measures.

Parameter	Outcome measures' usage			*p* value
	Does not use (%)	Use (%)	Total (%)	
	29 (27.6)	76 (72.4)	105 (100)	
Age				
20-24	4 (3.8)	12 (11.4)	16 (15.2)	0.226
25-29	13 (12.3)	17 (16.2)	30 (28.6)	
30-34	8 (7.6)	26 (24.8)	34 (32.4)	
35-39	3 (2.9)	13 (12.3)	16 (15.2)	
40-44	0 (0)	4 (3.8)	4 (3.8)	
45-49	0 (0)	1 (1)	1 (1)	
50-54	1 (1)	1 (1)	2 (1.9)	
55-59	0 (0)	2 (1.9)	2 (1.9)	
Gender				
Male	16 (15.2)	42 (40)	58 (55.2)	0.933
Female	13 (12.4)	34 (32.4)	47 (44.8)	
Level of education				
Bachelors	24 (22.8)	62 (59.1)	86 (81.9)	0.888
Masters	5 (4.8)	14 (13.3)	19 (18.1)	
Working experience				
1-4 years	14 (13.3)	24 (22.8)	38 (36.2)	0.198
5-10 years	13 (12.4)	36 (34.3)	49 (46.7)	
11-15 years	1 (1)	13 (12.4)	14 (13.3)	
16-20 years	1 (1)	2 (1.9)	3 (2.8)	
Above 20 years	0 (0)	1 (1)	1 (1)	
Type of facility				
Public	18 (17.1)	59 (56.2)	77 (73.3)	0.107
Private	11 (10.5)	17 (16.2)	28 (26.7)	
Weekly hours				
1-10 hours	2 (1.9)	1 (1)	3 (2.9)	0.279
11-20 hours	1 (1)	2 (1.9)	3 (2.9)	
21-30 hours	3 (2.8)	13 (12.4)	16 (15.2)	
31-40 hours	16 (15.2)	51 (48.6)	67 (63.8)	
Above 40 hours	7 (6.7)	9 (8.5)	16 (15.2)	
Number of stroke patients seen in a week				
1-10	23 (21.8)	34 (32.4)	57 (54.3)	0.013
11-20	4 (3.8)	24 (22.9)	28 (26.7)	
21-30	1 (1)	14 (13.3)	15 (14.3)	
31-40	0 (0)	2 (1.9)	2 (1.9)	
41-50	1 (1)	2 (1.9)	3 (2.8)	
Recommended outcome measures at facility				
No	25 (23.8)	30 (28.6)	55 (52.4)	0.0001
Yes	4 (3.8)	46 (43.8)	50 (47.6)	

## Data Availability

The raw data and entered data used and/or analyzed for this current study will be shared upon formal permission from the University of Health and Allied Sciences and the Ghana Physiotherapy Association.
